# Variable Expression Common Immune Deficiency: A Report of Five Cases

**DOI:** 10.7759/cureus.61397

**Published:** 2024-05-30

**Authors:** Abir Bouhamdi, Mounia Serraj, Mohamed Biaz, Mohamed Chakib Benjelloun, Bouchra Amara

**Affiliations:** 1 Pneumology Department, Hassan II University Hospital, University of Sidi Mohammed Ben Abdellah, Fez, MAR

**Keywords:** systemic granulomatosis, intravenous immunoglobulins (ivig), serum protein electrophoresis (spep), bronchial dilatation, variable common immune deficiency

## Abstract

We present five cases of common variable immunodeficiency (CVID), comprising three women and two men with a mean age of 23.8 ± 9.2 years. All our patients suffered from recurrent bronchopneumonitis, with complications of purulent pleurisy in two cases, requiring decortication in one case, and resulting in bronchiectasis in three cases. Digestive tract infections were observed in four patients, while two patients presented with ENT infections. One case was complicated by bacterial meningitis. All patients presented with global hypogammaglobulinemia, with CVID and granulomatous manifestation in one case. Treatment consisted of monthly immunoglobulin infusions.

## Introduction

Common variable immunodeficiency (CVID) is the most common primary humoral immunodeficiency (PHID), characterized by insufficient antibody production leading to hypogammaglobulinemia of variable intensity. This complex immunological disorder most often manifests itself in adulthood, with a remarkable diversity of clinical and immunological manifestations. Patients with CVID may present with a significant drop in immunoglobulin G (IgG), often accompanied by a decrease in IgA and/or IgM, as well as recurrent bacterial infections [1]. This disease represents a diagnostic challenge due to its heterogeneous nature and the need to exclude other causes of primary or secondary hypogammaglobulinemia.

CVID is a significant public health problem, although its exact prevalence remains difficult to determine due to geographical variations and potential underdiagnosis. However, studies estimate its prevalence at between 1 in 50,000 and 1 in 100,000 inhabitants [1,2], making it one of the most common primary immune deficiencies. The disease has a considerable impact on patients' quality of life, due to the high incidence of recurrent infections, pulmonary complications such as bronchiectasis, gastrointestinal manifestations, and other associated complications.

Diagnosis of CVID remains a clinical challenge due to its variable clinical presentation and the need to exclude other causes of hypogammaglobulinemia. The International Diagnostic Criteria (ICON) for CVID were revised in 2016 [3], but diagnosis often relies on a combination of clinical manifestations, laboratory findings, and immunological evaluation. In addition, the therapeutic management of CVID is complex and relies primarily on lifelong immunoglobulin-based replacement therapy.

In this study, we review five cases of adults with CVID, highlighting the extreme clinical and immunological variability of this disease. We hope that this analysis will contribute to a better understanding of the pathogenesis of CVID, improved diagnostic strategies, and more effective therapeutic approaches to improve the management of this complex disease.

## Case presentation

Case 1

Mrs. K.N., aged 28, has a history of bronchopneumonitis and chronic diarrhea since the age of 18, as well as three abortions linked to feto-maternal alloimmunization. Admitted for a persistent pyothorax despite two months of antibiotic treatment and chest drainage, she underwent a paraclinical workup revealing autoimmune hemolytic anemia and hypogammaglobulinemia on plasma protein electrophoresis. Immunoglobulin assays showed below-normal values. IgA = 0.25 g/l (reference range: 0.7-4 g/l), IgG = 3.2 g/l (reference range: 7-16 g/l), and IgM = 0.25 g/l (reference range: 0.4-2.3 g/l). Indirect immunofluorescence of lymphocyte subpopulations revealed low CD19 levels at 5 cells/ul. The diagnosis of CVID was made. The patient received regular immunoglobulin infusions with improvement of symptoms.

Case 2

Mr. B.H., aged 28, had a history of recurrent bronchopneumonia since the age of 21 and was admitted for a productive cough accompanied by signs of tuberculosis impregnation. Tests for Koch's bacilli in sputum and bronchial fibroaspiration fluid were negative. Chest computed tomography revealed images of bronchial dilatation associated with a focus on right lower lobar condensation and multiple mediastinal adenopathies. Differential diagnosis included hematopoietic and pulmonary tuberculosis and lymphoma. Mediastinoscopy revealed an epithelioid giganto-cellular granuloma without caseous necrosis. Abdominal ultrasound revealed 18 cm of homogeneous splenomegaly with hepatomegaly. Blood tests revealed anemia and lymphopenia, while immunoglobulin levels showed hypogammaglobulinemia. The diagnosis of systemic granulomatosis associated with CVID was made. The patient was put on regular infusions of immunoglobulin normal human polyvalent IV with good clinical improvement.

Case 3

**Figure 1:**
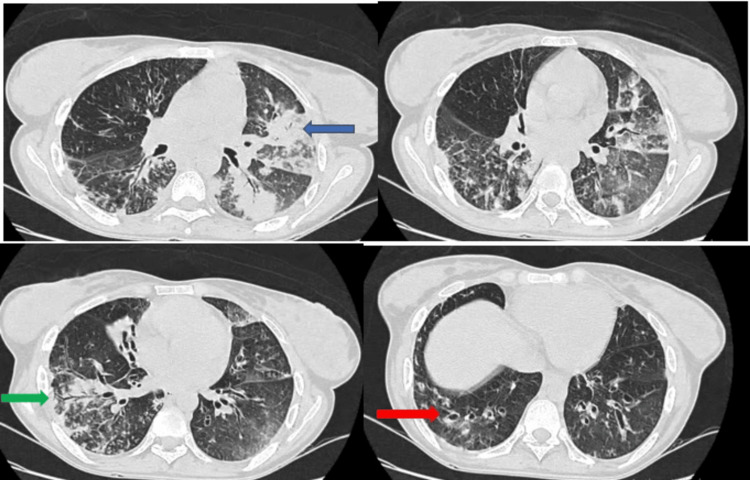
Axial-slice CT scans: foci of lung parenchymal condensation (blue arrow) surrounded by ground-glass areas, superimposed by peri-lobular reticulations associated with dilatations of cylidric (green arrow) and moniliform (red arrow) bronchi containing patchy mucoid impactions

Mrs. F.F., aged 42, has had a history of recurrent bronchopneumonia and ENT infections since the age of 35. She is admitted for acute hypercapnic respiratory failure due to superinfection of diffuse bronchiectasis, confirmed by a chest CT scan (Figure 1). Acute non-invasive ventilation, respiratory physiotherapy, and antibiotic therapy. Etiological work-up revealed hypogammaglobulinemia with IgA = 0.25 g/l (0.7 to 4), IgG = 3.2 g/l (7 to 16) and IgM = 0.45 (0.4 to 2.3). Lymphocyte phenotyping showed a deficient lymphocyte count: lym CD19+ = 14 cells/ul (72-414), lym CD3+ = 982 cells/ul (1000-2500), lym CD3+ CD4+ = 398 cells/ul (500-1000), lym CD3+ CD8+ = 456 cells/ul (200-600). The diagnosis of diffuse bronchiectasis at the stage of respiratory failure associated with a CVID was made. The patient is currently respiratory stable on near-monthly immunoglobulin infusion, cotrimoxazole antibiotic prophylaxis, respiratory physiotherapy, and home oxygen therapy.

Case 4

Mrs. F.F., aged 47, has had a history of bronchopneumonitis and digestive disorders since the age of 20, characterized by alternating episodes of diarrhea and constipation, associated with recurrent ENT infections. Admitted with an infectious picture, a thoracic CT scan revealed bilateral alveolar foci with a localized bronchiectatic image. Bronchoalveolar lavage cultures isolated *Klebsiella pneumoniae*, for which the patient was treated with ciprofloxacin. Gastrointestinal investigations, including oeso-gastro-duodenal fibroscopy and coproculture, were unremarkable. In view of the recurrence of clinical symptoms with infectious foci in different territories, several diagnoses were considered, including humoral immune deficiency, cryptogenic organized pneumonia, or systemic disease. A new bronchoalveolar lavage culture isolated *Pseudomonas aeruginosa*, and the patient was treated with a double dose of levofloxacin (500 mg*2 times per day). Two months later, the patient was readmitted to the hospital with purulent pleurisy, drained with appropriate antibiotic therapy. Paraclinical tests revealed iron-deficiency anemia and lymphopenia, while the immunological work-up was negative. Weighted immunoglobulin assay showed hypogammaglobulinemia, confirming the diagnosis of CVID. The patient was put on regular immunoglobulin infusions, cotrimoxazole-based antibiotic prophylaxis, combined with respiratory physiotherapy, with a good clinical course over a three-year follow-up.

Case 5

**Figure 2:**
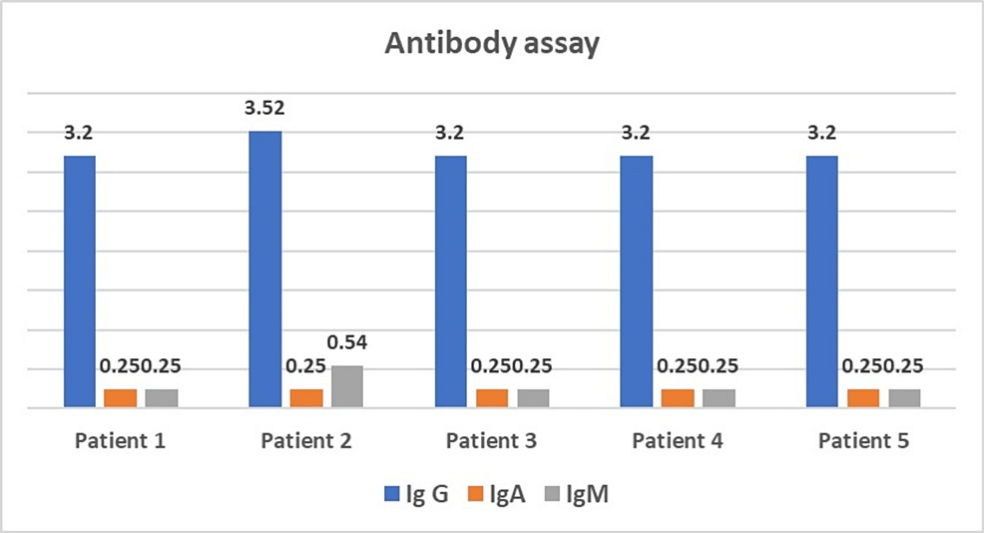
Antibody levels of the five cases

Mr M.Z., aged 28, presented with a history of recurrent bronchopneumonitis associated with episodes of liquid diarrhea since the age of four. At the age of 19, he began presenting with episodes of low-grade hemoptysis accompanied by morning bronchorrhea, which prompted his medical consultation. Investigations revealed localized bronchiectasis with an alveolar focus on chest CT. Bronchoalveolar lavage cultures revealed the presence of *Haemophilus influenzae* and pneumococcus, prompting the prescription of protected Amoxicillin. The alveolar focus was complicated by purulent pleurisy, which required subsequent decortication due to its encystment. Despite treatment, pneumonia recurred in the same pulmonary territory, prompting bronchial fibroscopy to rule out a local cause. An etiological work-up revealed hypogammaglobulinemia, with IgA = 0.25 g/l (normal: 0.7 to 4), IgG = 3.2 g/l (normal: 7 to 16), and IgM = 0.25 g/l (normal: 0.4 to 2.3) (Figure 2). The presence of hypoalbuminemia raised the suspicion of a secondary immune deficiency associated with malabsorption syndrome or celiac disease. An oeso-gastro-duodenal fibroscopy with biopsy revealed ileitis and subacute colitis, but serologies for celiac disease were negative. Eventually, the diagnosis of CVID was made, and the patient was treated with monthly infusions of Tegeline, resulting in a dramatic improvement in symptomatology after 10 courses. However, due to the lack of resources and one year after stopping treatment, the patient presented with an episode of bacterial meningitis complicated by a right hemi-corporeal tonic-clonic seizure, with a post-critical deficit of the right hemiplegic type (Table 1).

**Table 1:** Summary of the results of our observations

Patient	1	2	3	4	5
Age/sex	28/F	28/F	28/F	28/F	28/F
Onset of clinical signs (years)	18	21	35	20	4
Consanguinity	No	No	No	No	No
Respiratory symptoms	Recurrent bronchopneumonia Pyothorax	Recurrent bronchopneumonia Bronchial dilatation	Recurrent bronchopneumonia Bronchial dilatation Acute hypercapnic respiratory failure	Recurrent bronchopneumonia Bronchial dilatation Purulent pleurisy	Recurrent bronchopneumonia Bronchial dilatation Purulent pleurisy
ENT symptoms	No	No	Recurrent ear infections	Recurrent otitis and otorrhagia	No
Gastrointestinal manifestations	Liquid diarrhea	No	No	Alternating diarrhea and constipation	Liquid diarrhea
Organomegaly	No	Splenomegaly Hepatomegaly	No	No	No
IgG assay -g/l (7-16)	3.2	3.2	3.2	3.2	3.2
IgA assay -g/l (0.7-4)	0.25	0.25	0.25	0.25	0.25
IgM assay -g/l (0.4-2.3)	0.25	0.25	0.45	0.45	0.25
B lymphocytes CD9+ cells /ul (72-414)	5	-	14	-	-
CD3+ T lymphocytes /ul (1000-2500)	-	-	982	-	-
Lymphocytes T CD3+ CD4+ cellules /ul (500-1000)	-	-	398	-	-
T lymphocytes CD3+ CD8+ cells /ul (200-600)	-	-	456	-	-
Evolution	Clinical improvement with Tegeline	Clinical improvement with Tegeline	Clinical improvement with Tegeline	Clinical improvement with Tegeline	Clinical improvement but bacterial meningitis one year after stopping Tegeline

## Discussion

CVID is a rare condition characterized by a constitutional deficiency of humoral immunity. The ICON for CVID was revised in 2016 and complemented the guidelines established by the European (European Society for Immunodeficiencies (ESID)) and American (Pan American Group for Immunodeficiencies (PAGID)) learned societies [3]. To confirm the diagnosis of CVID, it is necessary to observe a significant drop in IgG and IgA levels, with or without a decrease in IgM. The IgG level must be less than two standard deviations from the mean (-2DS), in at least two measurements taken more than three weeks apart, unless it is initially very low (<1-3g/depending on age).

The presence of at least one of the following is also considered a potential indicator of CVID such as increased susceptibility to infection, autoimmune manifestations, granulomatous disease, polyclonal lymphoproliferation of unexplained origin, a family history of anticopr deficiency, inadequate vaccine response (and/or absence of isohaemagglutinins A or B) to at least one antigen, whether T-cell-dependent or T-cell-independent. However, it should be noted that the presence of the latter criterion is not systematic in all cases. Other diagnostic clues include a decrease in switched memory B cells (CD19+ IgM-/D- CD27+) below 70% of normal values for age or the absence of significant T-cell deficiency. It is imperative to exclude secondary causes of hypogammaglobulinemia, such as lymphoid hemopathies, as well as the use of drugs such as Rituximab, certain antiepileptics or antipsychotics like Clozapine, before making a definitive diagnosis.

The prevalence of CVID varies between studies, ranging from 1 in 50,000 to 1 in 100,000 inhabitants [1,2]. In our study, a female predominance was observed despite the small sample size, which is consistent with data in the literature. Several previous large-scale studies have reported a balanced gender distribution or a slight female predominance, with onset in men around the age of 20 and in women more likely around the age of 30 [4]. However, cases of CVID diagnosed in childhood or after the age of 70 have also been reported.

The diagnosis of CVID is usually made between the second and fourth decades of life. In our study, the mean age at diagnosis was 30 years, although the first symptoms of the disease can occur as early as the first years of life. In a previous series by Tahiat and colleagues [1], the mean age of onset of symptoms was 13.5 years, which is lower than in our study (19.6 years). In contrast, in a European series conducted by Chapel and colleagues [5], the first symptoms could appear at any age, with an average of 35 years. Consanguinity was noted in the series by Tahiat and colleagues in 13.8% of patients, which included a total of 29 patients.

Upper airway infections such as sinusitis, bronchitis, and pneumonia are the main presenting features of variable common immunodeficiency [6]. Bacterial pneumonia is by far the most frequent infectious complication, occurring in 63% to 86% of patients with CVID. In various studies, a high prevalence of pneumonia has been reported (86%) in the series by Hermans and colleagues [7] including 50 cases, 63% in the series by Hermaszewski and colleagues [8] involving 240 cases, 83% in the series by Cunningham-Rundles and colleagues [9] including 248 cases, and 100% in our own series.

*Streptococcus pneumoniae* and *Haemophilus influenzae* are the most commonly isolated pathogens [1]. A few cases of opportunistic infections, such as tuberculosis or infections with Mycobacterium spp, *Pneumocystis jirovecii*, or *Cryptococcus neoformans*, have also been reported in various series [10].

The recurrence of pulmonary infections favors the development of bronchiectasis (DDB). This complication was observed in 31% of patients in the Tahiat series, 34% of patients in the Italian series [11], and 60% in our own series.

The consistent finding of hypogammaglobulinemia across all cases is depicted in Figure 2. This figure shows the antibody levels (IgG, IgA, and IgM) for each patient, highlighting the hallmark of CVID-significantly reduced levels of immunoglobulins. This immunological deficiency underpins the clinical manifestations of recurrent infections and autoimmune complications seen in these patients.

Gastrointestinal manifestations are highly variable and frequent, occurring in 20-60% of patients, and can often be revelatory of CVID [12]. They are mainly characterized by chronic or recurrent diarrhea, observed in 58% of cases in the series by Hermans and colleagues, 18% in the series by Cunningham and colleagues, 12% in the series by Hermaszewski and colleagues, and 60% in our own series. This diarrhea is often multifactorial, resulting mainly from chronic infections, notably *Giardia lamblia*, but can also be attributed to specific lesions of the intestinal mucosa such as villous atrophies simulating celiac disease [3,6].

Other, albeit rarer, infections have also been reported, such as meningoencephalitis and osteoarticular tuberculosis [1]. In our series, a case of bacterial meningitis complicated by a tonic-clonic seizure was observed.

In 5-15% of cases of CVID, a granulomatous disease similar to sarcoidosis may develop, although the underlying mechanisms remain unknown. Immunoclinical associations have been noted, such as a decrease in CD4 counts, an inversion of the CD4/CD8 ratio, and an increase in CD8+CD57+ HLA-DR+ lymphocytes. A possible involvement of TNF in this syndrome is suggested by spontaneous hyperproduction of TNF by PBMCs. Moreover, this granulomatous disease seems to be more frequent in patients with abnormalities in the B lymphocyte population, such as an absence of switched memory B cells (M-27+) and an increased proportion of CD21low cells. In 40% of cases, sarcoid granulomatosis may be the first symptom, underlining the importance of performing plasma protein electrophoresis when diagnosing sarcoidosis [13,14].

The pathophysiology of CVID remains largely unknown. Only a minority of these deficiencies have an identified genetic origin. Genetic abnormalities have been observed, including a homozygous deletion in the ICOS gene, involved in cooperation between T and B lymphocytes, and mutations affecting the CD19 and TACI genes, essential for B lymphocyte differentiation and survival. Mutations affecting PI3Kα kinase have also been described, leading to autoimmune disorders and CD8+ T-cell dysfunction. Alterations in T-cell regulatory genes have also been reported [14].

Therapeutic management of CVID focuses on the assessment of complications, with particular emphasis on the risks of bronchial dystrophy (bronchial dilatation) and bronchial colonization by antibiotic-resistant germs. Respiratory physiotherapy is a key element in the management of bronchial suppurations.

Treatment of CVID relies primarily on lifelong replacement therapy, mainly with intravenous or subcutaneous polyvalent immunoglobulins. In a series of studies by Busse and colleagues [15], a six-year course of treatment reduced the frequency of annual episodes of pneumonia from 84% to 22%, with benefits as early as the first year, results similar to those observed in our own series. Although there is no standardized protocol, it is generally recommended to start with a bolus of intravenous immunoglobulin at a dose of 1 mg/kg, followed by either monthly intravenous infusions at a dose of 0.4 g/kg, or weekly subcutaneous infusions at a dose of 0.1 to 0.2 g/kg [16].

There are several limitations to this study. First, the sample size is relatively small, consisting of just five cases, which could hamper the generalizability of the results and the robustness of the conclusions. In addition, the study is based on a retrospective analysis of medical records, which may introduce data collection bias and compromise the reliability of the results. Furthermore, the lack of long-term follow-up of patients after treatment limits understanding of long-term outcomes and assessment of therapeutic efficacy.

Future directions

The pathophysiology of CVID involves a complex interplay between immune dysregulation and susceptibility to infections. Recent studies have highlighted the importance of immunological checkpoints, such as V-domain Ig suppressor of T cell activation (VISTA) and soluble PD-L1, in modulating immune responses. VISTA, for instance, acts as a mediator of immune quiescence and has shown promise as a target in cancer immunotherapy [17]. Soluble PD-L1 has been identified as an early marker of progressive disease, particularly in the context of immunotherapy [18]. Investigating these checkpoints in CVID could provide valuable insights into the mechanisms of immune dysregulation and open new avenues for therapeutic interventions. Understanding the role of these and other immune modulators could lead to more effective management strategies for CVID and improve patient outcomes.

## Conclusions

Our study highlights the diagnostic complexity and variability of clinical presentations of CVID, illustrating the importance of a holistic approach and multidisciplinary management of this disease. Advances in our understanding of its pathophysiology, as well as the development of more targeted therapeutic strategies, are essential to improve clinical outcomes and quality of life for patients with this condition. Early detection is crucial to reducing the morbidity and mortality associated with CVID. Unfortunately, in many cases, the diagnosis is made late, often at the stage of complications. This underscores the importance of raising clinicians' awareness of the clinical manifestations of CVID, particularly in the face of recurrent episodes of respiratory or gastrointestinal infections in adults.

In conclusion, close collaboration between clinicians, immunologists, and researchers is required to address the persistent challenges associated with the diagnosis and management of CVID, to optimize clinical outcomes, and to meet the unmet needs of this patient population.
